# An Efficient Group-Based Replica Placement Policy for Large-Scale Geospatial 3D Raster Data on Hadoop

**DOI:** 10.3390/s21238132

**Published:** 2021-12-05

**Authors:** Zhipeng Liu, Weihua Hua, Xiuguo Liu, Dong Liang, Yabo Zhao, Manxing Shi

**Affiliations:** School of Geography and Information Engineering, China University of Geosciences, Wuhan 430074, China; liuzhipeng@cug.edu.cn (Z.L.); huaweihua@cug.edu.cn (W.H.); bomer2000@sina.com (D.L.); zyaboedu@sina.com (Y.Z.); shimanxing@cug.edu.cn (M.S.)

**Keywords:** 3D raster, distributed GIS, Hadoop Distributed File System, replica placement

## Abstract

Geospatial three-dimensional (3D) raster data have been widely used for simple representations and analysis, such as geological models, spatio-temporal satellite data, hyperspectral images, and climate data. With the increasing requirements of resolution and accuracy, the amount of geospatial 3D raster data has grown exponentially. In recent years, the processing of large raster data using Hadoop has gained popularity. However, data uploaded to Hadoop are randomly distributed onto datanodes without consideration of the spatial characteristics. As a result, the direct processing of geospatial 3D raster data produces a massive network data exchange among the datanodes and degrades the performance of the cluster. To address this problem, we propose an efficient group-based replica placement policy for large-scale geospatial 3D raster data, aiming to optimize the locations of the replicas in the cluster to reduce the network overhead. An overlapped group scheme was designed for three replicas of each file. The data in each group were placed in the same datanode, and different colocation patterns for three replicas were implemented to further reduce the communication between groups. The experimental results show that our approach significantly reduces the network overhead during data acquisition for 3D raster data in the Hadoop cluster, and maintains the Hadoop replica placement requirements.

## 1. Introduction

Three-dimensional raster data have long been used to model continuous 3D spatial objects due to their simple representation and analysis [1,2]. With the rapid development of remote sensing technology, the integration of spectral and temporal information with spatial information helps to move from a 2D representation to 3D raster data structures [3]. The requirement for accuracy and resolution is increasing, evoking explosive growth in the amount of data obtained from these fields, easily reaching gigabyte, terabyte, and even petabyte scales [4]. It is difficult to analyze the increasing volume of geospatial 3D raster data under the traditional management and processing architecture.

Processing large-scale geospatial data in a distributed computing environment is becoming common practice [5,6]. Hadoop [7], an open-source big data framework applied to clusters of commodity hardware, is gaining increasing popularity in geoscience applications. Although Hadoop hides the complex distributed details and provides simplified parallel computing models, it is designed for general-purpose distributed storage and computing. Optimizations from different levels are often required for different spatial data analysis characteristics [4]. The rapidly increasing volume of 3D raster data needs many cluster resources, which makes the optimization very important. Related works for geospatial big data on Hadoop have mainly focused on parallel analysis and storage based on the original Hadoop; the storage mechanisms of Hadoop Distributed File System (HDFS) have not been modified; and the influence of data storage for spatial analysis is rarely considered. Optimizing Hadoop distributed storage for spatial data while satisfying the distributed storage requirements is a challenge.

In distributed environments, data storage locations usually have a significant impact on the performance of the parallel analysis of spatial data. Unfortunately, HDFS distributes the uploaded data in the cluster without considering its spatial characteristics. As a result, many distributed sub-tasks require data from multiple files placed on different datanodes. Massive network overhead is produced and degrades the performance of the cluster during the distributed analysis procedure. Network overheads often cause a bottleneck for parallel computing. In our practice, when distributed spatial operations run directly on the 3D raster data stored in the unmodified Hadoop environment, the remote data reading is the most time-consuming process in the parallel analysis.

**Figure 1:**
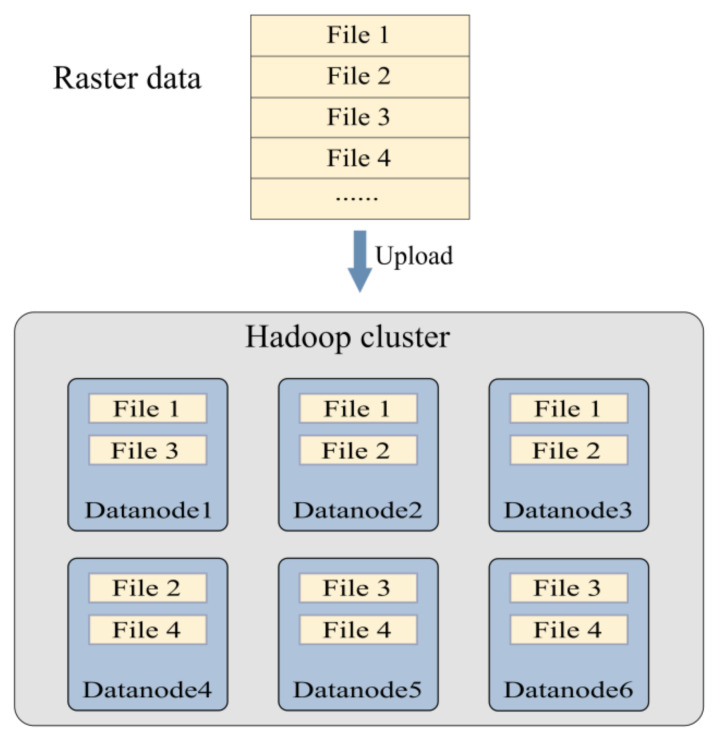
Possible raster data distribution on Hadoop cluster. The raster data are decomposed into rows for clarity.

To better describe the influence of replica placement for 3D raster data, a possible raster data distribution in the cluster through a simple division is depicted in Figure 1. The raster data are split into multiple files, and every file is further divided into one or more blocks, each of which has three replicas in the different datanodes. We assume each file is smaller than the block size and corresponds to one block in Figure 1. There are no datanodes for storing both File 2 and File 3. As a result, parallel sub-tasks requiring data from both files cannot be processed directly without data communication between two datanodes. This situation is common in large Hadoop clusters that store many files and seriously degrades the performance of the cluster, particularly those running bandwidth-sensitive applications. The data locality problem exists widely in distributed geospatial raster data analysis applications, especially for 3D raster data or 2D raster data with a large analysis range. In the analysis of 3D raster data, distributed analysis applications relying on a moving window often require multiple file blocks in a task, such as spatial filtering and resampling. For 2D raster data with a large analysis range, such as mapping and visualization tasks for a region, massive remote reading cannot be avoided and more cluster resources are required.

Some efforts have been implemented to process large-scale raster data in a distributed environment. General parallel programming models for raster data based on the Message Passing Interface (MPI) were designed in pRPL [8], which allows the raster analysis algorithm to be easily scaled to operate in distributed computing architecture. A strategy for raster-based geocomputation under CUDA, MPI and MPI/OpenMP was presented in [9]. Compared to traditional distributed computing frameworks such as Message Passing Interface (MPI), Hadoop provides a distributed file system and hides the details of the distributed availability and scalability. To provide efficient general remote sensing processing on Hadoop, a strip-oriented parallel programming model was implemented in [10]. A real-time big data analytical architecture for remote sensing data was proposed from aspects of a data acquisition unit, processing unit, and analysis decision unit in [11]. To efficiently retrieve massive ocean RS images, [12] modified the mean-shift algorithm for distributed processing in the Hadoop environment. In [13], a logical segmentation indexing approach was proposed to realize the distributed index for metadata of satellite images. A Spark-based parallel computing approach for the spatial neighboring analysis of terrain data was employed in [14]. The geospatial raster data are usually managed in accordance with their spatio-temporal dimension. To improve the query efficiency for three or more dimensional raster data, [15] designed a MapReduce system for spatio-temporal satellite data and, as such, a multi-resolution quad-tree based index structure was provided for the application of queries and visualizations. A spatio-temporal indexing approach was proposed for big climate data in [16]. A storage model for block-oriented HDFS and a grid partition algorithm were designed for efficient parallel processing based on the index. A hierarchical indexing strategy for Apache Spark with HDFS was implemented in [17]; a global k-d tree index and local hash table were provided to efficiently query and process the multi-dimensional raster data. Most of these works were built on the top of Hadoop without the modification of Hadoop components. Although the data locality problem has been alleviated through file organization in HDFS in some works, the default replica placement policy was deployed. Data were randomly distributed in the cluster, which may cause a large network overhead in the distributed analysis for 3D raster data. The programs pRPL [8] and XHAMI [18] redundantly stored neighboring data of the file region for every divided file, achieving low network overhead at the cost of disk storage space. However, the disk overhead increased significantly if the analysis algorithm required a large radius or multi-dimensional data were considered.

Besides the raster data distributed systems, general geospatial data processing frameworks based on Hadoop, such as Hadoop-GIS [19] and SpatialHadoop [20], provided multiple distributed spatial indexes and operations based on MapReduce. Based on SpatialHadoop, ST-Hadoop [21] designed a temporal hierarchy index structure for spatio-temporal queries, and injected spatio-temporal awareness inside each layer of SpatialHadoop. An integrated GIS platform architecture for spatio-temporal big data was described in [22], which contains the latest big data technologies based on cloud computing, 3D and virtual reality, container technology, and GIS systems. Apache Spark [23] was introduced to combine resilient distributed datasets (RDDs) [24] and Spark SQL [25] with spatial indexes to implement distributed in-memory spatial queries in the cluster. To provide a SQL-style spatial query framework, four categories of atomic grid transformations based on Apache Hive were designed in [26], and a complex climate analysis could be conducted by integrating these transformations. Some applications of spatial analysis in the Hadoop environment have been proposed, such as 3D spatial query engines [27] and spatio-temporal vehicle trajectory processing [28]. These studies on spatial data provide multiple implementations for the processing of spatial data in a distributed environment. However, the replica placements of spatial data were not considered, which may degrade the performance of geospatial 3D raster data analysis with data locality problems.

Data replication is generally used to improve the I/O efficiency and availability in distributed environments. It is usually necessary to design different replica placement strategies for different applications. With the default replica placement policy, three replicas are placed under two racks, finding a balance between writing efficiency and reliability. In [29], data management and replication approaches were surveyed based on the performance metrics of availability, scalability, fault tolerance, load balancing, throughput, and consistency. Storage locations were optimized through a consistent hashing-like algorithm in [30], and a parallel join query algorithm with a scheduling strategy was designed. To improve the data locality problem caused by random replica placement, a lightweight extension of Hadoop was proposed to store related data on the same datanode in [31]. Spatial relationships among uploaded files were ignored in these studies. CoS-HDFS [32] colocated spatially related files based on minimum bounding rectangles in the same datanode. However, all divided raster data files are contiguous and related; thus, it is difficult to directly place all related data in a single datanode using these methods. In the current research for data locality optimization on Hadoop, three replicas of the colocated files have the same colocation patterns. Our method implements different colocation patterns for three replicas through the overlapped group scheme; thus, the data locality can be further improved.

There are usually two ways to handle the data locality problem. The first option is to design more complex parallel operations to ensure that each sub-task receives only the intermediate result from the remote datanodes instead of the original data. Should the communication of the intermediate result be very small, as it usually is, the network overhead can be significantly reduced. However, parallel operations need to be designed according to different analysis methods, and the complex processes may lead to greater programming difficulties and more potential errors. The second option is to optimize the replica locations of HDFS, which allows the data required by each sub-task to be placed in the same datanode as much as possible. We chose the latter option in this paper, which facilitates the implementation of the parallel analysis and scales the traditional methods to the distributed environment.

The data locality problem exists widely in distributed geospatial raster data analysis applications, especially for multi-dimensional raster data or applications with a large analysis radius. Related research improved the data locality at the cost of additional data storage, and three replicas of HDFS were not considered to form different colocation patterns. To the best of our knowledge, this is the first work that investigates the optimization of replica placement for geospatial raster data on Hadoop. In this paper, we propose an efficient static replica placement policy of HDFS optimized for large-scale geospatial 3D raster data, mainly focusing on the problem of a large network overhead and load balancing in the analysis of an entire region. An overlapped group scheme is designed and different colocation patterns for three replicas are implemented through the scheme. The data locality of parallel tasks is obviously improved and the distributed storage requirements of HDFS are also maintained. Our method is suitable for typical geospatial raster data analysis of batch processing with the data locality problem, especially for 3D raster data or 2D raster data with a large analysis range. Since most spatial parallel analysis sub-tasks can be performed with less network overhead through our method, high concurrency scenarios without hot regions are also applicable.

The rest of this paper is organized as follows. Section 2 describes the main methods of our group-based data placement policy. The experiments and a discussion of the results are provided in Section 3 and Section 4. Finally, Section 5 provides some conclusions regarding this study.

## 2. Materials and Methods

### 2.1. Group Method of 3D Raster Data

HDFS is a highly fault-tolerant distributed file system designed to run on commodity hardware. Typically, a master server called namenode manages the metadata of the file system and regulates access to files by clients, and many server machines called datanodes are responsible for the data storage. When a file is uploaded onto HDFS, the file is split into one or more blocks and stored in a set of datanodes. Each block of a file has multiple replicas in different datanodes for fault tolerance and read efficiency. The number of replicas is usually set to three, which is the number that is mainly utilized in our method, although this can be set higher with few modifications.

For geospatial raster data, access to a sub-region of entire data is a common operation; thus, it usually splits all the data into many files according to the spatial grid. Compared to storing the entire data on Hadoop, splitting all the data into grid files is more efficient for sub-region data acquisition. With our method, geospatial 3D raster data are divided into grid files and uploaded to the HDFS. Our designed replica placement policy chooses three datanodes for every divided file, which is finally stored in these datanodes if no errors are detected. The main idea of the group method is to divide the raster data into overlapped groups, and one replica set of divided files in each group is placed in a datanode to reduce the communication within the group. Communication among groups is further reduced through the overlapping area of groups. For clarity, we describe the optimized group method used for 2D raster data from a column-based group method to a grid-based group method, and then extend the method to 3D raster data.

#### 2.1.1. Column-Based Group Method for 2D Raster Data

**Figure 2:**
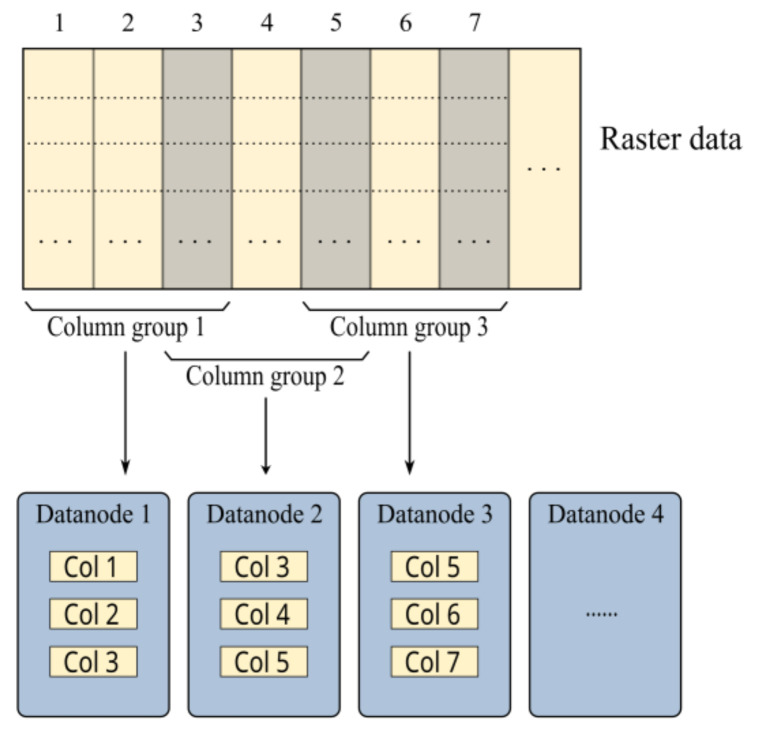
Example of the column-based group method for 2D raster data. Data are first divided into many grid files, files in every three columns are assigned to a column group, and one replica set of these files is placed on a datanode. “Col 1” in figure represents all grid files in the first column.

Two-dimensional raster data are usually decomposed into rows, columns, or grids for distributed processing. In our column-based group method, the grid files in every several columns were assigned to a group (three columns in Figure 2), and one replica set of these files was placed on a datanode. An overlapping column was set between every two adjacent groups. To maintain the storage balance in the cluster, replicas that were not assigned were distributed randomly with the consideration of the storage status of the datanodes.

The spatial width corresponding to each divided file was usually greater than twice the analysis radius. Therefore, the entire region could be processed without data communication between groups, and data locality could be guaranteed by two replicas. However, the number of columns and groups was small in some cases, which made it difficult to distribute the computations evenly to more nodes. The following grid-based group method will show how this problem was solved.

#### 2.1.2. Grid-Based Group Method for 2D Raster Data

**Figure 3:**
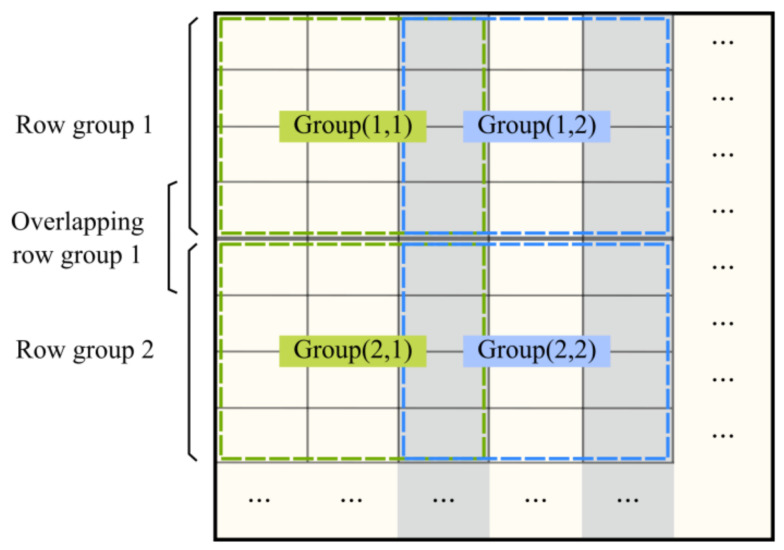
Example of grid-based group method for 2D raster data. The group method for every row group is similar to the column-based group method. One replica set of files in every normal group and overlapping row group is assigned to a datanode.

Based on the column-based group method, we first grouped X × Y divided files (4 × 3 in Figure 3) instead of several columns. Groups in each row formed a row group, and the internal processing of a row group was the same as the above column-based group method. The grid files in each row group were assigned to overlapping normal groups such as “group (1, 1)” and “group (1, 2)” in Figure 3. To avoid network communication among the row groups, the boundary files of every two adjacent row groups were placed into a new overlapping row group, which was used to analyze the boundary region between every two row groups. With this method, the grid files were assigned to normal groups and overlapping row groups, and one replica set of the files in each group was assigned to a datanode.

The number of files in the overlapping row group was often much larger than that of the normal group, but only a small part of these files were used for processing, and the problem of an imbalanced computing load did not occur in practice.

#### 2.1.3. Extension to 3D Raster Data

For 3D raster data, each group consisted of *X* × *Y* × *Z* 3D grid files, and groups with the same z-coordinate range were placed into a layer group and processed in the same way as 2D raster data. Although it was difficult to avoid network overhead between every two layers, the size of group *Z* could be set larger in order to limit the number of layers, and the network communication would be much smaller than the previous overhead. The horizontal range of geospatial 3D raster data was usually much larger than the vertical range in geospatial applications, thus the number of layers in the z-direction was generally small.

### 2.2. Parallel Analysis for Group Method

In the above data placement method, every group was assigned to a datanode, and a set of replicas of the group were placed in the node. Analysis within each group could then be independently processed by the corresponding datanode. Through an overlap of groups in the row and column directions, data transfer between groups in the two directions could be avoided. To minimize the data transmission overhead, the analysis area for each datanode needed to be allocated according to the group placement.

**Figure 4:**
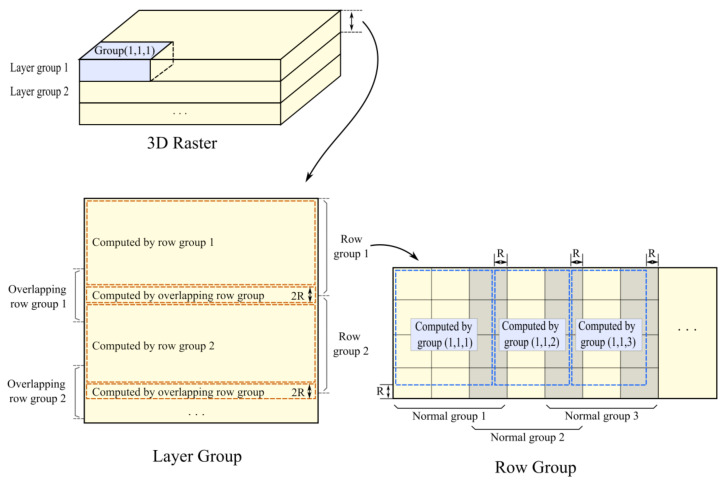
Assignment of analysis region for every group based on optimized data placement. In row groups, each normal group is responsible for the region that can be analyzed within the group data. For each layer group, regions that are not allocated by the normal groups are assigned to the overlapping row groups.

Details of the analysis region for each group are shown in Figure 4. In every row group, normal groups were assigned computing areas in order. Each column group was responsible for an unallocated area that could be processed without data communication with the other groups. In layer groups, areas between two adjacent row groups were computed by overlapping row groups. As an overlapping row group consisted of more files than the other groups, the computed height was set to 2 R to avoid an imbalanced computing load. For multiple layer groups, a data transmission with a distance of R in adjacent layers was difficult to avoid and produced the main network overhead in our method.

### 2.3. Implementation of Group-Based Replica Placement Policy

To achieve storage based on the above policy, there are two methods for uploading divided files to proper datanodes. The storage locations of all files can be scheduled before the upload, and the HDFS uses the prescheduled information to place the data block of every file directly [30]. Another method is to determine the storage location when uploading files, in which the group information of previously uploaded files is recorded in a table (called a group placement table) and used for dynamically determining the placement of the current uploading file. The latter method is used in this study owing to its ability to obtain the replica placement dynamically according to the latest status of the datanode.

**Figure 5:**
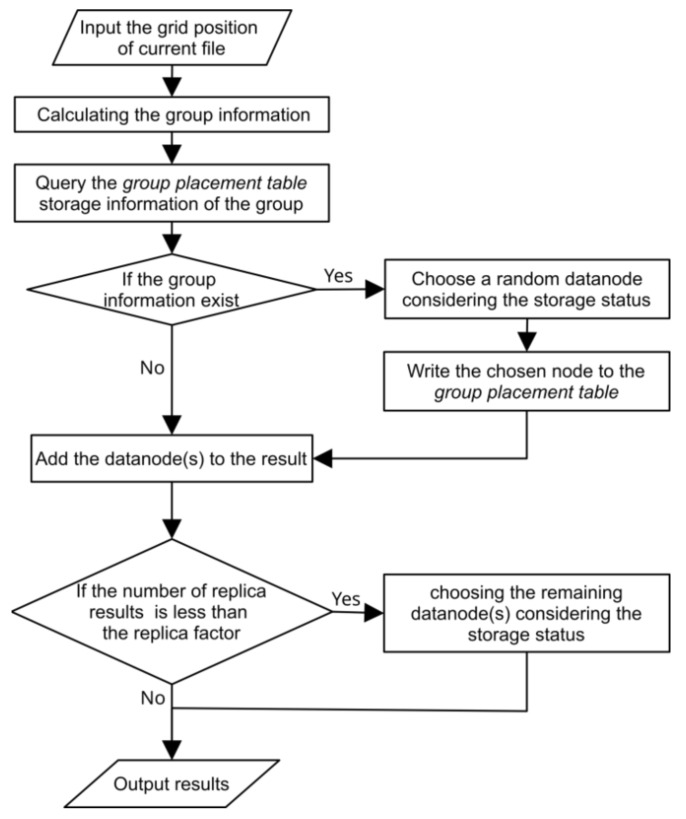
Flowchart for optimized method of chooseTarget. Storage locations of divided grid files are chosen considering the group information and storage status of cluster.

The 3D raster data are first divided into regular 3D grid files. BlockPlacementPolicy is a component of HDFS for controlling the placement of replicas. The default Hadoop block placement policy is optimized for the 3D raster data based on the group-based method. The divided 3D grid files are uploaded in x-order. For every divided file, the core method chooseTarget is invoked and returns the appropriate data storage information according to the group placement and cluster status. The optimized method of chooseTarget is illustrated by the flowchart in Figure 5, which includes the following steps:

STEP 1: Input the grid position of the current file.
STEP 2: Calculate the group information for the current uploading divided file based on the grid position (the file may belong to multiple groups).
STEP 3: Querying the group placement table to obtain the storage information of each group.
STEP 4: If no group record is queried, then choose a random datanode from the cluster by considering the storage status of the datanodes and the position of the previous replicas, adding the chosen datanode to the replica result, and writing the storage information into the group placement table.
STEP 5: If the storage information is queried, then add the datanode in the query to the replica result.
STEP 6: If the number of replica results is less than the replica factor (default 3), then choose the remaining datanode(s) randomly from the cluster by considering the storage status.
STEP 7: Return the chosen results for the storage locations of the current input file.

Some details are worth consideration if we are to improve the storage performance. Regarding the write efficiency and data reliability, the HDFS rack policy is applied to place three replicas of each file into two racks (in Step 5). To maintain the storage and computing balance of the cluster, information in the group placement table is further considered to distribute groups evenly into the cluster when selecting a random datanode for the group (in Step 3). The number of blocks stored in each datanode is also recorded to maintain the storage balance by controlling the random datanode selection for the file (in Step 5).

After the data are uploaded, the data placement may be affected by the failure of the datanode or the balancer utility, and the locations of the groups need to be considered in the data movement. The group records of the unreachable datanode in the group placement table are reassigned first when the abnormal node is detected, and the new datanode is chosen using a similar block placement policy. In the balancing process, blocks of 3D raster data are moved individually considering existing groups, and the groups are reorganized according to the requirements of analysis.

## 3. Results

This section describes an extensive experimental study conducted to evaluate the performance and data reliability of the above algorithm. Our group-based replica placement policy places as many adjacent data in the same datanode as possible, thereby obtaining better data locality compared to the default data placement policy in theory. Three experiments were conducted to prove its efficiency. In these experiments, the impact on the default features of HDFS was first verified. The IO efficiency of our method was then compared with the colocation-based replica placement policy extended from CoS-HDFS [31] and the default replica placement policy. The effect of different group parameters was finally analyzed.

### 3.1. Experimental Setup

#### 3.1.1. Experiment Environment

Cluster Setup. We evaluated our system by running experiments on a cluster of 11 KVM (Kernel-based Virtual Machine) machines that consisted of one namenode and ten datanodes. Each node had three cores (at 2.2 GHz), 16 GB of memory, and a 100 GB hard drive. All nodes ran CentOS 7.2, Java 8, Hadoop 2.7, and Spark 2.1. In the HDFS configurations, the default block size was set to 128 MB, and three racks were simulated for four, three, and three datanodes in each rack, respectively.

Datasets. We employed a 3D raster geological model of the Huangtupo slope built from boreholes, sections, detailed geological maps, and a digital elevation model to assist the geological hazard analysis, located on the south bank of the Yangtze River in Badong, Hubei, China. The model contains 1600  × 2400 ×  800 voxels, and each voxel contains properties of stratum type (1 byte), lithology (1 byte), moisture content (1 float), uncertainty indicator (1 float), and hazard indicator (1 float) for a total of approximately 40.1 GB. All data were divided into 8 × 12 × 4 files and then uploaded to the cluster, and the properties of the voxels in each file were stored in binary format without compression. The size of each divided file was less than the block size of HDFS, thus avoiding further division of the files into multiple blocks by HDFS.

Although the experiment employed the data and application from our geological project, common storage format and analysis operations were employed and can be extended to other 3D raster applications.

#### 3.1.2. Description of Parallel Analysis of Comparative Methods

To improve the data locality of raster data analysis, rRPL [8] and XMIDI [18] redundantly store neighboring data of the file region for every divided file. The two models achieved a low network overhead at the cost of disk storage space, which is hard to compare with our optimized method. To the best of our knowledge, there are no studies directly optimizing replica placement for raster data on Hadoop. Therefore, we extended the colocation-based idea of CoS-HDFS in the manuscript to 3D raster data as a comparative approach for IO efficiency. Additionally, the default replica placement policy of Hadoop was also used as another comparative method. Different parallel approaches can be used for the replica placements. In this experiment, we designed three parallel analytical strategies for the data uploaded through the two comparative methods and our group-based approach.

For the default replica placement policy, the region of every divided file is treated as a computing unit, which reads data from the region and surrounding area within a certain radius. Parallel analysis for every computing unit is conducted after the data reading. The implementation extends Hadoop’s InputFormat class to create computing units. Every computing unit is assigned to an InputSplit, which stores the spatial information of the corresponding region. A datanode that stores more data in the region is chosen for the computation. RecordReader generates a key-value pair for each InputSplit, in which the key represents the ID of the unit, and the value represents the data required for analysis.

The colocation-based method colocates the group files based on CoS-HDFS. All 3D raster data are divided into contiguous groups, and files in every group are colocated and have the same replica placement through the extension of CoS-HDFS. Unlike our method, every 2 × 3 × 2 files are grouped, and there is no overlap between these groups. The same group size as our method is used for comparison. Every group with its surrounding area is assigned to a computing unit, in which data are divided into key-value pairs for parallel analysis by Apache Spark.

Our group-based policy uses a similar approach for parallel analysis. The main difference is that our group method divides the entire data into overlapped groups, which reduces the network overhead among groups. Every group is treated as a computing unit instead of a file region, and the datanode placing the group is chosen for analysis.

It is worth noting that some implementations can achieve a higher efficiency, but these methods are more complex and may cause higher difficulty in programming as well as possible errors. The bandwidth efficiency does not improve significantly as the data locality is not changed.

Parallel processing can be achieved through MapReduce and Apache Spark, the latter of which was chosen for our experiment, owing to its efficient in-memory computing model. A parallel analysis of each InputSplit was conducted through a map procedure, and the results were merged during the reduce procedure.

### 3.2. Impact on Default Features of HDFS

The purpose of the default rack-aware replica placement policy is to improve data reliability, availability, and network bandwidth utilization. Considering the writing efficiency and reliability of the data, three blocks of each file should be placed in three datanodes under two racks. In addition, the computational load of every datanode is approximately proportional to the number of groups, and the storage load is proportional to the number of file blocks. Therefore, blocks and groups should be distributed evenly in the cluster to maintain the load balance.

Three-dimensional raster data divided into 8 × 12 × 4 files (for a total of 384) were uploaded 10 times through the HDFS API, the statistical information of which (such as the max/min/average of the data distribution information mentioned above) is shown in Table 1.

For the rack rule of HDFS, 46.4 files on average were not distributed under two racks, with the number varying between 32 and 60. All of these files were in the overlap area of three groups (two normal groups and one overlapping row group). These files were still distributed on three datanodes, and most of them were placed in three racks, which had little effect on the performance of the upload or the reliability of the data.

Through data balance optimization of the uploaded files and groups, all groups and files were allocated to ten datanodes evenly. The difference between the group number of every two datanodes was less than two. Limited by the rack rule of files, some datanodes had up to four more placed blocks than the other datanodes.

### 3.3. Evaluation of Network IO Performance

Resampling is a typical data-intensive 3D raster analysis operation. A distributed resampling method for the uploaded 3D raster data was first employed to evaluate the network read performance. Then, a geological analysis based on resampling was performed to prove the applicability. In the resampling method, the new value of a cell was determined based on the surrounding voxels within a radius, and three radii (7, 10, and 13) were used in the resampling experiments. A group size of 2 × 3 × 2 was used in the optimized method.

We focused on the network overhead caused by a 3D raster of the data read procedure during the experiment, which can be obtained from the IO statistics of HdfsDataInputStream in RecordReader. The read network overhead was used as the major indicator that directly reflects the locality of the data, which determines the efficiency of bandwidth-intensive jobs and is less influenced by the analysis method and hardware performance of the cluster. The running time increased with an increase in the read network overhead in our experiments. As the overlapped area of the computing units may be read multiple times in different datanodes, the read overhead of the disk is also listed as a comparative indicator of efficiency.

**Figure 6:**
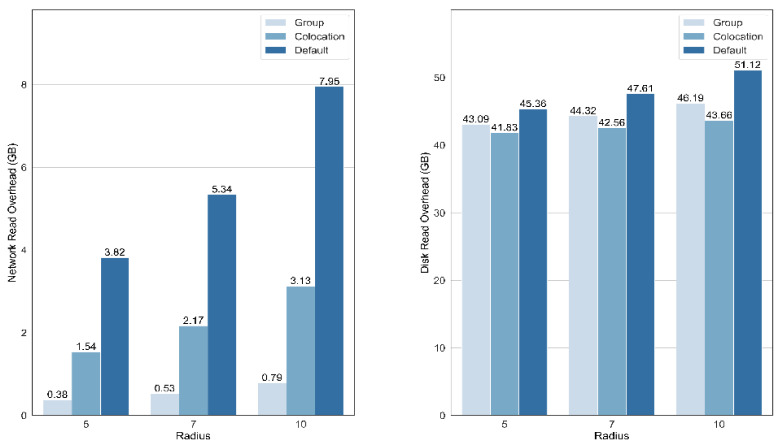
IO performance of the group-based method, the colocation-based method, and the default Hadoop replica placement policy. Network and disk read overheads are compared among these methods.

Figure 6 shows the read statistics of the resampling algorithm for the three replica placement policies. The results show that our group-based method produces a significant reduction in the network read overhead. For the same resampling radius, the amount of network read for data uploaded by our method is approximately 10% of the default replica placement policy and approximately 25% of the colocation-based approach. There was a small decrease in disk data read compared to the default replica placement policy, indicating that the repeated disk read overhead for the overlapped area is relatively small. For the overlap between groups in our method, more groups were generated than the colocation-based method; thus, the disk overhead was slightly higher.

**Figure 7:**
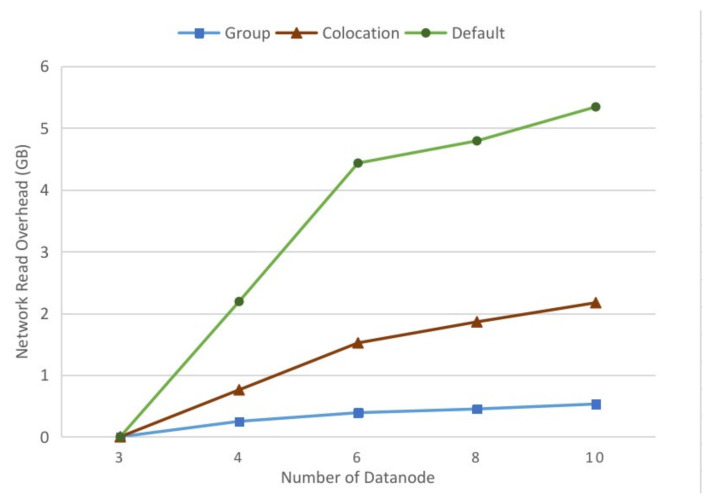
IO performance of the group-based method, the colocation-based method, and the default Hadoop replica placement policy with different numbers of the cluster nodes.

To evaluate the performance in a more complex distributed environment, different numbers of cluster nodes were employed in the experiment. With the group size of 2 × 3 × 2 and the radius of 7, the number of cluster nodes was set to 3, 4, 6, 8 and 10. Figure 7 shows the read statistics of different numbers of the datanodes in the cluster. When the cluster consisted of only three datanodes, every datanode stored the replicas of all divided files, and all parallel tasks could obtain the required data locally and the network read overhead of data reading could be ignored. The network read overhead increased with the increase in the number of cluster nodes. The amount of network read for data uploaded by our method is approximately 10% of that of the default replica placement policy and 20% to 30% of that of the colocation-based approach. The optimization of our methods became more obvious with the increase in cluster nodes.

Hadoop is designed to run on clusters of commodity hardware, and the datanode failure is common in the large cluster. Therefore, the performance of datanode failure was evaluated. When the simulated datanode failure was detected by the namenode, the distributed resampling job was submitted to the resource manager. The radius of the resampling was set to 7, and the group size was set to 2 × 3 × 2. In this situation, tasks that should be operated on the failed datanode were migrated to other datanodes, with the most essential data stored. The network read overhead of the group-based method was increased from 0.53 GB to 1.02 GB, which was 19% of the default replica placement policy and approximately 47% of the colocation-based approach, and operated without failure. The disk read overhead was the same as that of the previous job that operated without failure. After the replicas of the failed datanode were recovered from the remaining datanodes, the new submitted job achieved a similar result to those of the cluster with 9 nodes.

### 3.4. Effect of Group Size

**Figure 8:**
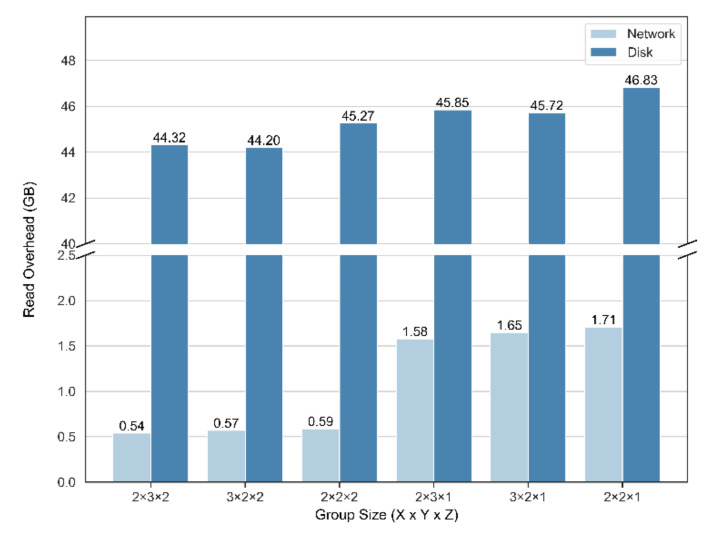
Statistics of read overhead for different group sizes in the group-based method. Overhead was significantly greater when group height was 1. With the same group height, group length and width had less impact on read overhead.

The group size determines the size of every storage and analysis unit in our method, which is the main parameter of this algorithm. For a radius of 7, we recorded the read overhead of the group sizes of 2 × 3 × 2, 3 × 2 × 2, 2 × 2 × 2, 2 × 3 × 1, and 3 × 2 × 1 (Figure 8). For convenience, we used length × width × height to represent the group size in the x-, y-, and z-directions.

The network and disk overheads produced by group sizes of 2 × 3 × 1, 2 × 2 × 1, and 3 × 2 × 1 during the reading procedure were significantly greater than those in the other groups, and the impact of the network overhead was greater than that of the disk. With our method, which is limited to the number of replicas in the HDFS, the remote data reading of every group in the z-direction is difficult to avoid through the overlapping of groups. The increase in the group number in the z-direction will directly lead to a larger data exchange among groups in different z layers.

When the group height was the same, the group length and width had a lower impact on the read overhead. This is due to the fact that the required data for every computing unit in the x- and y-directions were placed in the same datanode, and thus the influence of the network read was mainly caused by a data exchange in the z-direction.

### 3.5. Case Study: Geological 3D Raster Data Analysis

Our method provides an optimization of replica storage locations in the cluster, most applications with data locality problems can benefit from our approach. To prove the applicability, a typical 3D raster data analysis scenario from the geological hazard analysis application is introduced. By analyzing the geometric and geological information contained in the 3D raster model, a hazard probability model was obtained to find the region requiring more attention for disaster prevention or further geological surveys. In order to obtain higher analysis accuracy and shorter analysis time, Hadoop was introduced to process the large-scale 3D raster data instead of the traditional non-distributed method. When default replica placement policy was applied in the analysis, data acquisition was the most time-consuming process and had a serious impact on the IO performance of other applications running on the cluster.

**Figure 9:**
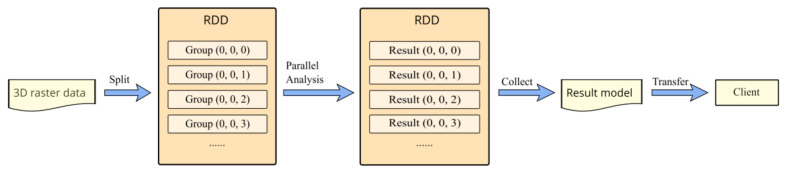
Workflow of a parallel geological analysis for 3D raster data through the group-based method. In the colocation-based and the default Hadoop replica placement, corresponding data splitting methods are used instead of the group-based division.

To make our experimental results applicable to other applications, we chose a typical 3D raster data analysis scenario from the geological hazard analysis application. For commonly used parallel raster analysis, the main difference in our case is that the hazard analysis was performed in each task. The parallel analysis procedure is shown in Figure 9. The 3D raster data is first split into multiple groups according to our optimized replica placement policy. Hazard analysis in each group is performed simultaneously in the corresponding datanodes, which can be replaced with other applications. In every region of 10 × 10 × 5, the probability of hazard is calculated from the geometric shape of the surrounding stratum type and lithology. A weighted sum of the probability and the uncertainty indicator is computed as the result of the region. After the parallel analysis through Spark RDD, the results are collected from the RDD of the worker nodes in the cluster. Then, the 3D raster result model is transferred to the client computer for further analysis and visualization. Since every 10 × 10 × 5 region corresponds to a new voxel value in the result 3D raster model, the resulting model is small enough for direct processing in the client.

**Figure 10:**
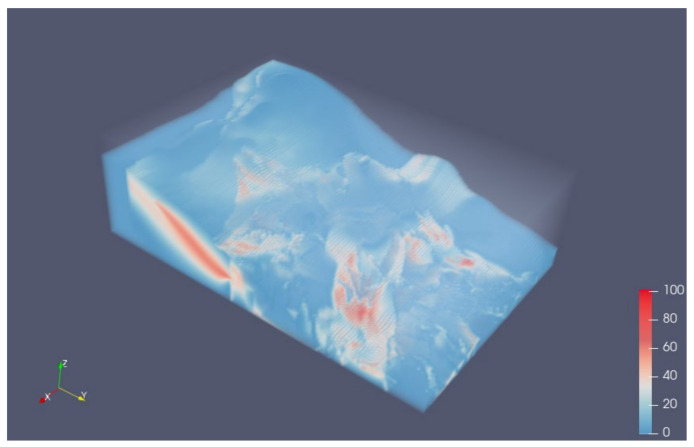
Rendering images of geological hazard analysis result model. The red areas in the figure indicate a high probability of hazard in our analysis method, further analysis in these areas were required.

Volume rendering through the Visualization Toolkit (VTK v9.1.0, https://vtk.org/ accessed on 20 September 2021) was used to visualize the result 3D raster data. The rendering image is shown in Figure 10. The voxel value represents the hazard probability of the corresponding location according to the analysis method. Through the resulting model, we could identify the regions which required more attention as being hazardous. The red areas in the figure were marked as warning areas, which were inferred by the unstable geological structure or lack of survey data. The origin model and geological survey data in these areas were further analyzed, and an interactive analysis was conducted to determine if disaster prevention or further geological survey was required.

After uploading the 3D raster data, there are two large-scale analysis requirements: parallel analysis for the entire region of the raster data, and analysis jobs for parts of the raster data submitted from multiple clients. In the parallel analysis of the entire region, ignoring the overhead of the collect and transfer procedure, similar results were acquired as in previous experiments. The remote network read was reduced to 11% of the default replica placement policy. The average job running times through the group-based, colocation-based and default replica placement policy were 15 min, 24 min, and 31 min, respectively. The main reason for the long run times of the two comparative methods is the relatively low remote read efficiency of HDFS. HDFS short-circuit local reads were configured in the cluster, allowing the client to read local data directly and boost the read performance. Therefore, tasks with better data locality showed better performance. The remote read overhead of the comparative methods can be alleviated through a more complex design of RDD iteration. However, these methods lead to great difficulty in programming, and extra efforts for the parallel procedure are required for different analysis methods of 3D raster data.

Owing to the optimization of replica placement in the datanodes, our method also showed a similar performance improvement in multiple analysis jobs for parts of the raster data. Each sub-task read the most required data locally if the analysis jobs were evenly distributed throughout the entire region. The load balance problem occurred when a hot region existed in the analysis jobs, which could not be solved by our method and the compared methods. Further optimizations such as tuning the locations, caches, and replica numbers, would improve the performance.

## 4. Discussion

To ensure availability and writing efficiency, HDFS places three replicas of each file under two racks. In our method, each overlapping row group overlaps with many normal groups, and it is difficult to make all the files belonging to the three groups meet the rack condition. Only 8–16% of the data files broke the default rack rule of HDFS, and most of these files were placed under three racks, which had little effect on the writing efficiency. Few files were placed on three datanodes of the same rack, increasing the risk of data reliability from rack failures. However, this slight influence can be avoided by further optimizing block placement.

Specific replica placement strategies should be designed for different applications. Our method is designed for the 3D raster data of batch processing and concurrency scenarios without hot regions, and it is important to maintain the load balance in the distributed analysis of an entire region. Every group corresponded to a computing unit in our method, and the groups were placed evenly across the datanodes in the experiment; thus, tasks ran locally with load balance in the distributed analysis. To ensure the efficiency of parallel analysis, when the data in the cluster were unevenly distributed, the datanodes with less usage were not given priority to store the groups. Instead, we improved the load balance of storage by optimizing the locations of the replicas that did not affect the locality of the groups. The storage load balance was sacrificed to improve the efficiency of batch processing in the entire region.

In the experiment, we compared the network IO performance of three replica placement policies. With the default replica placement policy, the network read overhead is mainly produced by the remote reading of the surrounding data that are not stored on the current computing node. Divided files are randomly distributed in the cluster, resulting in a large amount of data exchange among the datanodes. The colocation-based replica placement policy reduces network communication by simply placing the replicas of adjacent files on the same datanode. However, network overhead between groups cannot be avoided. Our method uses a group overlap scheme to decompose the analysis of a group layer into some local tasks, and the network overhead is mainly produced in data acquisition from different group layers.

In a Hadoop cluster, the namenode periodically receives a heartbeat from each datanode to obtain the storage information. A datanode is considered a failure after the heartbeats are lost in a set period (over ten minutes by default). When this occurs, the block manager of the namenode instructs the remaining datanodes to create additional replicas for the lost blocks. The block recovery procedure in the block manager can be modified for the group-based replica placement, and the blocks of each group in the failed datanode are created in the same datanode. After the recovery procedure, the storage state of groups is similar to that of the cluster with one less node.

Generally, the more data a single task processes, the less extra cost it incurs. However, processing a large amount of data in a single task reduces the degree of parallelism, making it difficult to take full advantage of large-scale computing resources and may also lead to problems such as the load balance of long tasks and insufficient memory resources for a single task. In our practice, network overhead is mainly produced between layer groups; thus, a larger group height can be used for better IO performance. The group length and width can be set according to the degree of parallelism. If the group length and width are set to smaller values, more computing units and greater IO overhead will be produced. When the computing resources provided by the cluster are insufficient for there to be a high degree of parallelism, the group height can be set to equal the number of divided files in the z-direction, which is degraded to a 2D grid-based group method, and data communication among the computing units can be avoided.

A geological analysis application was introduced to prove the applicability of our method, and a similar result was obtained. Our method is an optimization for the storage layer on Hadoop. Neighborhood-based parallel analyses such as resampling and spatial filtering perform a similar data read procedure. Therefore, the data locality problem in different applications can be alleviated through our optimized replica placement policy. Since our method distributes the 3D raster data evenly in the cluster, the load balance problem may occur when a hot region exists in the analysis jobs. Under these circumstances, further optimizations for data storage and caches according to the hot region are required.

Based on our optimized replica placement, different parallel analysis methods can be designed. The experiments showed an example of typical parallel 3D raster data analysis through Apache Spark. First, the optimized replica placement policy was implemented on Hadoop, which allowed the raster data to be placed as the optimized group-based method. Next, parallel task partitioning for the optimized storage was implemented. In Apache Spark, the task partitioning was realized through the implementation of Hadoop’s InputFormat class. Then, traditional non-distributed 3D geospatial analysis methods were applied in each task simultaneously. Finally, the results of the tasks were collected, which could be transferred to the client to perform further analysis through traditional 3D raster analysis software, or used for additional parallel analysis. In addition to the improvement of data locality, the parallel analyses can be simplified by our parallel task partitioning model, which allows the non-distributed geospatial raster analysis method to be easily migrated to each task and run concurrently.

## 5. Conclusions

Geospatial 3D raster data have been widely used for continuous 3D objects and 2D satellite images with spectral and temporal dimensions. The spatial information of the uploaded files is not considered in the replica placement of Hadoop, and this results in high communication overhead and significantly influences the cluster performance. In this study, we proposed a novel group-based replica placement policy for the batch processing of geospatial 3D raster data, intending to reduce the network overhead caused by files of adjacent regions randomly placed on multiple nodes.

The geospatial 3D raster files were first arranged into overlapped groups, in which the divided files were placed in the same datanode. The storage locations of the replicas were further optimized to reduce the communication between groups through an overlap. The experimental results show that our algorithm has little effect on the writing efficiency and reliability of the HDFS; the storage and computing load balances are well maintained; and network overhead during data acquisition is significantly reduced. Comparative experiments of different group sizes were provided to better adjust the degree of parallelism according to the actual situation.

In a future study, we plan to further investigate the job scheduling method for different types of geospatial data analysis based on the current method. Moreover, the study of a data placement strategy for unevenly distributed vector data and complex data access schemes would be of interest.

## Figures and Tables

**Table 1:** Statistical information of 3D raster data distribution in ten uploads.

	Min	Max	Average
Number of files placed in one rack	2	12	6.8
Number of files placed in three racks	30	48	37.0
Number of files breaking the rack rule	32	60	46.4
Number of groups in datanodes	6	7	6.2
Number of blocks in datanodes	113	117	115.1

## Data Availability

Not applicable.
